# Prediction the changes of anthropometric indices following a weight-loss diet in overweight and obese women by mathematical models

**DOI:** 10.1038/s41598-024-65586-0

**Published:** 2024-06-24

**Authors:** Vahideh Ebrahimzadeh Attari, Mahdieh Nourmohammadi, Mohammad Asghari-Jafarabadi, Sepideh Mahluji, Aida Malek Mahdavi, Parya Esmaeili

**Affiliations:** 1 Department of Clinical Nutrition, Faculty of Nutrition and Food Sciences, Tabriz University of Medical Sciences, Tabriz, Iran; 2 Department of Nutrition and Food Sciences, Maragheh University of Medical Sciences, Maragheh, Iran; 3 Nutrition Research Center, Tabriz University of Medical Sciences, Tabriz, Iran; 4 Cabrini Research, Cabrini Health, Malvern, VIC 3144 Australia; 5 School of Public Health and Preventive Medicine, Faculty of Medicine, Nursing and Health Sciences, Monash University, Melbourne, VIC 3004 Australia; 6 Department of Psychiatry, School of Clinical Sciences, Faculty of Medicine, Nursing and Health Sciences, Monash University, Clayton, VIC 3168 Australia; 7 Road Traffic Injury Research Center, Tabriz University of Medical Sciences, Tabriz, Iran; 8 Tuberculosis and Lung Disease Research Center, Tabriz University of Medical Sciences, Tabriz, Iran; 9 Connective Tissue Diseases Research Center, Tabriz University of Medical Sciences, Tabriz, Iran; 10 Department of Epidemiology and Biostatistics, Faculty of Health, Tabriz University of Medical Sciences, Tabriz, Iran

**Keywords:** Anthropometry, Body size, Mathematical formula, Low-calorie diet, Obesity, Nutrition

## Abstract

Estimating the change rates in body size following the weight loss programs is very important in the compliance of those programs. Although, there is enough evidence on the significant association of body weight change with the other anthropometric indices and/ or body composition, there is so limited studies that have depicted this relationship as mathematical formulas. Therefore, the present research designed to use a mathematical model to predict changes of anthropometric indices following a weight-loss diet in the overweight and obese women. In this longitudinal study, 212 overweight/obese women who received an individualized low-calorie diet (LCD) were selected and followed-up for five months. Anthropometric measurements such as weight, waist circumference (WC), hip circumference (HC), and body composition (lean mass and fat mass) were performed. Then, body mass index, waist to hip ratio (WHR), waist to height ratio (WHtR), a body shape index (ABSI), abdominal volume index (AVI), and body adiposity index (BAI) were calculated using the related formula. Following the LCD led to the substantial and consistent changes in various anthropometric indices over time. All of these anthropometric variations were significantly related with the percent change (PC) of body weight except than WHR. Moreover, according to the mathematical formulas, weight loss was closely related to the decrease of WC (PC-WC =  − 0.120 + 0.703 × PC-WT), HC (PC-HC =  − 0.350 + 0.510 × PC-WT), body fat percentage (PC-Body Fat =  − 0.019 + 0.915 × PC-WT), WHtR (PC-WHtR =  − 0.113 + 0.702 × PC-WT), and improvements in ABSI (PC-ABSI =  − 0.112 + 0.034 × PC-WT) and AVI (PC-AVI =  − 0.324 + 1.320 × PC-WT). The decreasing rates of WC, HC, body fat percentage, WHtR, ABSI, and AVI in relation to the weight loss were clinically and statistically significant. This means that a healthy weight lowering diet would be accompanied by decreasing the body fat, body size and also the risk of morbidities.

## Introduction

Obesity is defined as an excessive body fat mass which can increase the risk of other chronic diseases^1^. The global prevalence of obesity has increased considerably in recent decades that makes it as an important healthcare challenge^1^. Based on World Health Organization’s report in 2022, about 2.5 billion adults aged ≥ 18 years were overweight and of these, 890 million were obese^2^. The prevalence of overweight and obesity is generally higher in females than males^2^. Obesity has a multifactorial etiology, but sedentary lifestyle and improper dietary patterns are proposed as two major contributing factors^3^. Therefore, the calorie restriction and increasing physical activity are suggested as the first step in most of weight loss programs^4^. Evidence shows that a modest weight loss (5–10% of body weight) can be accompanied by a general improvement in other comorbidities like hypertension (HTN) and type 2 diabetes mellitus (DM)^5^.

The body mass index (BMI) is widely used for assessing general obesity; however, it cannot assess body fat distribution^6^. Up to now, multiple anthropometric indices related with adiposity and its distribution have developed including waist circumference (WC), waist to hip ratio (WHR), waist to height ratio (WHtR), a body shape index (ABSI), abdominal volume index (AVI), and body adiposity index (BAI). It is indicated that WC is a valuable measurement, which has more relation with abdominal or visceral fat mass (VF) and can better predict obesity-related health risks (e.g., insulin resistance, hyperlipidemia, HTN, type 2 DM, and cardiovascular disease)^7,8^. Furthermore, hip circumference (HC) provides a more specific measure of subcutaneous gluteofemoral adipose tissue^9^.

A significant positive correlation was demonstrated between body weight changes and WC and/or HC in some previous randomized clinical trials (RCTs) with different interventions on the obese individuals^10–20^. A recent study showed that body weight, BMI, WC, and HC decreased significantly after a calorie-restricted high-protein diet, and intermittent fasting^10^. A significant reduction in body weight and fat mass (FM) was also indicated in obese or overweight subjects after adherence to both Mediterranean diet (MD) and the Very Low-Calorie Ketogenic diet (VLCKD), whereas the MD caused a higher reduction in WC and FM^11^. In other studies, a greater reduction in body weight, WC, and FM was noticed in obese participants following the VLCKD compared to the low-calorie diet^12,13^. Moreover, a time-restricted eating intervention caused a significant decrease in body weight in overweight men and women; however, a significant reduction in VF and WC was only observed in men^14^. Furthermore, body weight, BMI, and WC decreased significantly after adherence to the intermittent fasting diets^16^. The results of a more recent RCT showed that dietary restriction of carbohydrate and calorie reduced body weight, WC, and body fat in overweight/obese individuals^18^.

Although, the changes of body weight and WC have been aligned with each other in most of studies, this may not be always observed, especially in the middle-aged and older individuals who experience some body fat redistribution^21^. According to the results of Yuan et al.^22^, the increasing age was related with weight loss but also with increasing WC in middle-aged and older adults.

Reducing visceral fat and body size, particularly waist circumference (WC), in obese individuals is paramount for enhancing both physical and mental well-being, as well as sustaining motivation for continued treatment. Essential to this endeavor is the accurate estimation of changes in body size following weight-loss programs, a factor crucial for ensuring patient compliance. While ample evidence exists regarding the significant association between body weight change and other anthropometric indices or body composition, there remains a scarcity of studies delineating this relationship through mathematical formulas. Recent attention in medical research has turned toward leveraging mathematical models to deepen our understanding of disease dynamics and patterns^23^. These models offer invaluable assistance in crafting personalized treatment plans tailored to individual patient backgrounds and disease severity. By precisely delineating disease type and severity, mathematical models empower physicians to select optimal treatment strategies, thereby enhancing treatment satisfaction and patient outcomes. Consequently, mathematical modeling assumes a pivotal role in advancing medical research and clinical practice^23^.

To the best of our knowledge, there was only one study^24^ that reported the analytic correlation between weight loss and WC change (% Weight loss = 0.85 × Waist reduction (cm) − 2.09; r = 0.79). The present study aims to utilize a mathematical model to accurately predict changes in anthropometric indices resulting from a weight-loss intervention among overweight and obese women. Emphasizing the application of mathematical modeling, this research seeks to offer practical insights into the dynamics of body weight reduction, focusing on the interplay between dietary interventions and anthropometric outcomes.

## Methods

### Study population

This longitudinal study was performed on 212 overweight and/or obese women who referred to the obesity clinic affiliated to the Tabriz University of Medical Sciences, Tabriz and Maragheh University of Medical Sciences, Maragheh, Iran. The inclusion criteria were: the women age 18–60 years and having BMI 25–35 kg/m^2^. The exclusion criteria were: pregnancy and lactation; smoking; performing heavy physical activity; subjects with only one visit; any history of cardiovascular disease, type 2 DM, depression, cancer, kidney, liver or thyroid diseases; using a weight-loss diet in the past six months; taking weight-lowering medications, oral contraceptives, anti-depressants, and nutritional supplements over the past six months and during the study. The experimental protocol was approved by the licensing committee of Maragheh University of Medical Sciences, Maragheh, Iran and all methods were performed in accordance with the relevant guidelines and regulations. Also, all eligible participants were made aware of the study procedures and signed an informed written consent.

### Study design

Data about demographic characteristics and physical activity level were collected at baseline. For each participant, an individualized low-calorie diet was provided by an experienced dietitian. Resting energy expenditure (REE) was calculated based on the Mifflin equation^25^, and then energy for physical activity level and thermic effect of food were added to REE. After calculation of the required energy for each participant, a weight-loss diet plan was provided based on 500 kcal less than calculated energy along with considering their caloric intake level. The diet contained 50% carbohydrates, 20% protein, and 30% fat of total energy. All participants were followed-up monthly and were advised to continue low-calorie diet and other necessary changes were made if needed.

### Anthropometric and body composition measurements

The body weight was determined with light clothing without shoes on a calibrated Seca weight scale, accurate to 0.1 kg (Seca 762; Vogel & Halke, Hamburg, Germany). The body height was measured without shoes, by a standard tape with 0.1 cm precision. BMI was calculated using the following formula: weight (kg)/height (m^2^). The WC was measured in a standing position at the narrowest point between the lowest rib and the iliac crest using an elastic tape with 0.1 cm precision, whereas the HC was measured as the greatest gluteal circumference. Then, WHtR, WHR, ABSI, AVI, and BAI were calculated using the related formula as follow^26,27^:

- WHtR = WC/Height
- WHR = WC/HC
- ABSI = 1000*WC*Weight^−2/3^*Height^5/6^
- AVI = (2*(WC*100)^2^ + 0.7*(WC*100 − HC*100)^2^)/1000
- BAI = HC/((height)^1.5^ − 18)

Body composition consists of total body fat percentage (Body Fat), total body FM, total body fat free mass (FFM), and total body water was assessed by an 8-electrode bioelectrical impedance analyzer, accurate to 0.1 kg (Tanita BC-418 MA; Tanita Co., Tokyo, Japan), using the standardized protocol of device. All measurements were taken every 4 weeks (in monthly visits) by the same examiner.

### Statistical analysis

The data were analysed using Stata (Version 18.0, StataCorp, College station, Texas, USA). Descriptive statistics, including mean and standard deviation, and frequency and percentage were used for quantitative and qualitative variables, respectively. Normality of the data was confirmed by examining skewness and kurtosis indices. The changes in body measurements over time were calculated using change percent (PC) of each variable with respect to its baseline measure mean using formulae: PC = (value − mean_at_baseline)/(mean_at_baseline)*100.

The association among the changes in body measurements over time including ABSI, AVI, BAI, FFM, Body Fat, FM, VF, HC, WC, WHR, WHtR, and BMI as the outcomes, and the weight loss as the main independent variable were modelled using linear mixed model. Additionally, we adjusted for age to account for potential age-related variations in anthropometric changes. For the models we considered a compound symmetry covariance structure for the relationship among measurements over time. The restricted maximum likelihood estimation procedure was utilized to estimate the regression parameters and their 95% confidence interval (CIs). This analogy provided us with writing the regression equation for each index for the change percent of each variable. Additionally, post hoc tests were conducted using the Sidack correction for multiple comparisons. All results were considered significant when *P* < 0.05.

## Results

In current study, a total of 212 overweight and/or obese women were followed-up for five months. The baseline mean ± standard deviation (SD) of age, weight, BMI, WC, and HC were 38.07 ± 11.30 years, 84.43 ± 12.68 kg, 32.97 ± 4.79 kg/m^2^, 101.45 ± 10.45 cm, and 117.35 ± 9.51 cm, respectively.

### Anthropometric and body composition changes over time

Table 1 presents trends in various body measurements over time. Across the five months follow-up, the mean values and SDs for different variables were shown. Accordingly, weight consistently declined during the five months, reflecting the effectiveness of LCD. Moreover, other notable trends include a relatively stable ABSI over time, a gradual decrease in the BAI and AVI, a declining trend in BMI, and a consistent decrease in Body Fat. FFM appears to increase slightly, while FM fluctuates. HC and VF both demonstrate a steady decrease. WC decreases over time, as does the WHR. The WHtR also exhibits a downward trend.

**Table 1:** The anthropometric measurements of participants during weight loss program.

Indices	Time (months)										PC*		*P* value^#^
	1		2		3		4		5				
	Mean	SD	Mean	SD	Mean	SD	Mean	SD	Mean	SD	Mean (SD)	Median (min : max)	
ABSI	0.78	0.05	0.78	0.05	0.78	0.05	0.77	0.05	0.78	0.05	− 0.38 (6.01)	− 0.42 (− 17.68 : 17.47)	< 0.001
AVI	21.02	4.25	20.15	4.03	19.76	3.97	18.5	3.52	17.64	3.45	− 5.42 (19.48)	− 9.35 (− 44.89 : 51.09)	< 0.001
BAI	− 17.94	0.01	− 17.94	0.01	− 17.94	0.01	− 17.95	0.01	− 17.95	0.01	0.01 (0.03)	0.01 (− 0.12 : 0.32)	< 0.001
BMI	32.97	4.79	32.02	4.65	31.66	4.63	30.33	4.28	29.32	4.48	− 3.68 (14.38)	− 5.68 (− 33.12 : 45.18)	< 0.001
Body fat	42.92	6.72	41.78	6.56	41.46	6.52	39.82	6.32	38.6	6.67	− 3.30 (15.57)	− 5.42 (− 36.46 : 45.35)	< 0.001
FFM	24.04	2	24.26	2.05	24.57	2.21	25.09	2.1	25.45	2.05	1.88 (8.83)	0.66 (− 22.64 : 33.10)	< 0.001
FM	45.33	4.69	44.33	4.89	47.3	44.09	42.19	5.11	48.14	53.42	− 0.09 (58.07)	− 1.61 (− 43.08 : 1114.70)	< 0.001
HC	117.35	9.51	115.36	9.38	114.68	9.57	110.83	14.28	109.44	9.16	− 2.28 (8.95)	− 2.86 (− 98.47 : 26.97)	< 0.001
VF	8.82	2.38	8.19	2.34	7.72	2.22	6.99	2.28	6.36	2.19	− 10.15 (27.48)	− 9.27 (− 65.98 : 70.11)	< 0.001
WC	101.45	10.45	99.31	10.15	98.29	10.12	94.94	9.2	92.85	9.3	− 2.84 (10.21)	− 4.39 (− 27.06 : 24.20)	< 0.001
WHR	0.86	0.06	0.86	0.06	0.86	0.06	1.35	5.06	0.85	0.06	7.10 (213.77)	− 0.31 (− 20.89 : 5873.72)	< 0.001
WHtR	0.63	0.07	0.62	0.07	0.61	0.07	0.59	0.07	0.58	0.07	− 2.87 (11.28)	− 5.08 (− 30.61 : 27.24)	< 0.001
WT	84.43	12.68	81.98	12.32	81.31	12.1	77.76	10.48	74.9	11.02	− 3.62 (14.61)	− 4.95 (− 35.57 : 53.97)	< 0.001

*ABSI* a body shape index, *AVI* abdominal volume index, *BAI* body adiposity index, *BMI* body mass index, *BODY FAT* body fat percentage, *FFM* fat-free mass, *FM* fat mass, *HC* hip circumference, *VF* visceral fat, *WC* waist circumference, *WHR* waist-to-hip ratio, *WHtR* waist-to-height ratio, *WT* weight or body weight, *SD* standard deviation.

*PC: The percent change with respect to baseline measures calculated as: (value − mean_at_baseline)/(mean_at_baseline)*100.

^#^Based on repeated measure analysis of variance with Greenhouse–Geiser correction for sphericity.

### Association of the anthropometric measurements with weight loss over time

Table 2 displays the outcomes of a mixed model analysis for the association of body measurements with weight loss over time. Significant associations were observed for ABSI (B = 0.034; 95% CI 0.015, 0.052; *p* < 0.001), AVI (B = 1.320; 95% CI 1.279, 1.361; *p* < 0.001), BAI (B =  − 0.002; 95% CI − 0.002, − 0.001; *p* < 0.001), Body Fat (B = 0.915; 95% CI 0.909, 0.921; *p* < 0.001), FFM (B =  − 0.438; 95% CI − 0.489, − 0.387; *p* < 0.001), FM (B = 0.598; 95% CI 0.251, 0.945; *p* = 0.001), HC (B = 0.510; 95% CI 0.475, 0.545; *p* < 0.001), VF (B = 2.393; 95% CI 2.276, 2.510; *p* < 0.001), WC (B = 0.703; 95% CI 0.684, 0.721; *p* < 0.001), WHtR (B = 0.702; 95% CI 0.683, 0.721; *p* < 0.001), and BMI (B = 0.992; 95% CI 0.985, 0.998; *p* < 0.001) with weight loss across the study duration. However, the results were not significant for WHR (B =  − 0.077; 95% CI − 1.130, 0.975; *p* = 0.885) Furthermore, adjusting for age in the analysis had minor changes in the associations observed between the changes in body measurements over time and weight loss as the main independent variable. This suggests that age had minimal impact on the overall associations identified in our study (Table 2).

**Table 2:** The association of anthropometric measurements with weight loss over time.

	Unadjusted		Age adjusted	
	*B* (95% CI)	*P* value*	B (95% CI)	*P* value*
ABSI	0.034 (0.015, 0.052)	< 0.001	0.032 (0.013, 0.050)	0.001
AVI	1.320 (1.279, 1.361)	< 0.001	1.310 (1.269, 1.350)	< 0.001
BAI	− 0.002 (− 0.002, − 0.001)	< 0.001	− 0.002 (− 0.002, − 0.001)	< 0.001
BMI	0.992 (0.985, 0.998)	< 0.001	0.991 (0.985, 0.998)	< 0.001
Body Fat	0.915 (0.909, 0.921)	< 0.001	0.914 (0.908, 0.920)	< 0.001
FFM	− 0.438 (− 0.489, − 0.387)	< 0.001	− 0.437 (− 0.488, − 0.387)	< 0.001
FM	0.598 (0.251, 0.945)	0.001	0.605 (0.259, 0.951)	0.001
HC	0.510 (0.475, 0.545)	< 0.001	0.510 (0.475, 0.544)	< 0.001
VF	2.393 (2.276, 2.510)	< 0.001	2.217 (2.104, 2.329)	< 0.001
WC	0.703 (0.684, 0.721)	< 0.001	0.699 (0.681, 0.717)	< 0.001
WHR	− 0.077 (− 1.130, 0.975)	0.885	− 0.077 (− 1.130, 0.976)	0.886
WHtR	0.702 (0.683, 0.721)	< 0.001	0.699 (0.680, 0.717)	< 0.001

*B* regression coefficients, *CI* confidence interval, *ABSI* a body shape index, *AVI* abdominal volume index, *BAI* Body adiposity index, *BMI* body mass index, *BODY FAT* body fat percentage, *FFM* fat-free mass, *FM* fat mass, *HC* hip circumference, *VF* visceral fat, *WC* waist circumference, *WHR* waist-to-hip ratio, *WHtR* waist-to-height ratio, *WT* weight or body weight.

*Based on the linear mixed model analysis and the Sidack correction test as the post hoc test.

### The mathematical formulas for the relationships

The given set of equations represents mathematical formulas that express the change percent (PC) in various body measurements as a result of the change in body weight. Each equation follows a linear model structure, indicating the relationship between the change in body weight and the resulting change in the respective body measurement. Table 3 presents these mathematical formulas that show the rate and direction of change in different body measurements regarding the changes in body weight. According to the mathematical formulas, weight loss was closely related to the decrease of WC (PC-WC =  − 0.120 + 0.703 × PC-WT), HC (PC-HC =  − 0.350 + 0.510 × PC-WT), Body Fat (PC-Body Fat =  − 0.019 + 0.915 × PC-WT), BMI (PC-BMI =  − 0.024 + 0.992 × PC-WT), WHtR (PC-WHtR =  − 0.113 + 0.702 × PC-WT), and improvements in ABSI (PC-ABSI =  − 0.112 + 0.034 × PC-WT) and AVI (PC-AVI =  − 0.324 + 1.320 × PC-WT). In addition, weight loss was related to the minimal decrease in BAI (PC of BAI = 0.002 +  − 0.002 × PC-WT). Moreover, weight loss was closely related to the increase of FFM (PC of FFM =  − 0.568 +  − 0.438 × PC-WT). In addition, adjusting for age had little impact on the mathematical formula used to model the associations among the changes in body measurements over time and weight loss as the main independent variable. This indicates that the mathematical model remained robust even after accounting for age (Table 3).

**Table 3:** Mathematical formulas represents the percent changes of anthropometric measurements based on the weight loss.

Mathematical formulas	
Unadjusted	Age adjusted
PC-ABSI = − 0.112 + 0.034 × PC-WT	PC-ABSI = − 7.997 + 0.032 * PC_WT
PC-AVI = − 0.324 + 1.320 × PC-WT	PC-AVI = − 21.459 + 1.310 * PC_WT
PC-BAI = 0.002 + − 0.002 × PC-WT	PC-BAI = 0.034 + − 0.002 * PC_WT
PC-BMI = − 0.024 + 0.992 × PC-WT	PC-BMI = − 8.780 + 0.991 * PC_WT
PC-Body Fat = − 0.019 + 0.915 × PC-WT	PC-Body Fat = − 28.496 + 0.914 * PC_WT
PC-FFM = − 0.568 + − 0.438 × PC-WT	PC-FFM = 2.034 + − 0.437 * PC_WT
PC-FM = 2.856 + 0.598 × PC-WT	PC-FM = 15.015 + 0.605 * PC_WT
PC-HC = − 0.350 + 0.510 × PC-WT	PC-HC = − 3.505 + 0.510 * PC_WT
PC-VF = 2.064 + 2.393 × PC-WT	PC-VF = − 62.842 + 2.217 * PC_WT
PC-WC = − 0.120 + 0.703 × PC-WT	PC-WC = − 11.684 + 0.699 * PC_WT
PC-WHR = 6.817 + − 0.077 × PC-WT	PC-WHR = 32.430 + − 0.077 * PC_WT
PC-WHtR = − 0.113 + 0.702 × PC-WT	PC-WHtR = − 16.081 + 0.699 * PC_WT

*ABSI* a body shape index, *AVI* abdominal volume index, *BAI* body adiposity index, *BMI* body mass index, *BODY FAT* body fat percentage, *FFM* fat-free mass, *FM* fat mass, *HC* hip circumference, *VF* visceral fat, *WC* waist circumference, *WHR* waist-to-hip ratio, *WHtR* waist-to-height ratio, *WT* weight or body weight.

PC: change percent with respect to baseline measures calculated as: (value − mean_at_baseline)/(mean_at_baseline)*100.

## Discussion

Lifestyle changes including dietary interventions have been demonstrated to be an effective strategy for weight loss^28,29^. A proper and favorable dietary intervention seems not only cause a significant reduction in body weight but also leads to a meaningful decrease in body size, which indicates attenuation in FM. Furthermore, in order to motivate overweight/obese individuals for weight loss during nutrition counselling, it is important to inform them about the body weight and body size variations.

The results of this study revealed that following the LCD caused the substantial and consistent changes in various anthropometric indices over time, with Time1 as the reference category for comparison. According to our findings, body weight and BMI showed a significant and consistent decline. The HC, WC, and VF values consistently decreased, indicating a general decline in body size. Similarly, decreasing the AVI, ABSI, and BAI values over time suggested some improvements in the health-related measurements, body shape and also body composition. It should be mentioned that all of these anthropometric measurements’ variations were significantly related with the PC of body weight except than WHR. Generally, these findings provide valuable insights into the dynamic changes in anthropometric measurements over the study period.

Our study was consistent with some previous researches that evaluated the effects of different interventions on the obese individuals and showed significant positive correlation between body weight changes and WC and/or HC^10–20^. A recent study showed that body weight, BMI, WC, and HC decreased significantly after a calorie-restricted high-protein diet, and intermittent fasting^10^. A significant reduction in body weight and FM was also indicated in obese or overweight subjects after adherence to both Mediterranean diet (MD) and the Very Low-Calorie Ketogenic diet (VLCKD), whereas the MD caused a higher reduction in WC and FM^11^. Moreno et al.^12,13^ also indicated a greater reduction in body weight, WC, and FM in obese participants following the VLCKD compared to the low-calorie diet. In another study, Domaszewski et al.^14^ reported that a time-restricted eating intervention caused a significant decrease in body weight in overweight men and women; however, a significant reduction in VF and WC was only observed in men. Furthermore, body weight, BMI, and WC decreased significantly after adherence to the intermittent fasting diets^16^. In a study on severely obese women, significant greater weight loss and reduction of BMI, WC, and FM were reported in the low-carbohydrate diet group compared to the conventional-diet group^17^. These researchers concluded that WC measurements were sensitive to weight reduction^17^. In addition, the results of a more recent RCT showed that dietary restriction of carbohydrate and calorie reduced body weight, WC, and body fat in overweight/obese individuals^18^. According to the results of Rothberg et al.^19^, following a weight management program for six months, significantly decreased BMI and WC and a larger relative decrease in WC was associated with greater improvements in the components of metabolic syndrome. According to Salas-Salvadó et al.^20^ study, an intensive weight loss intervention led to significant weight loss and WC reduction in overweight/obese adults. Similarly, Iłowiecka et al.^30^ reported that all anthropometric indices including BMI, ABSI, and FM significantly reduced in obese individuals after the 12-month balanced energy-restricted diet. Furthermore, an individualized balanced low-calorie diet during eight weeks resulted in a remarkable decrease in body weight, BMI, WC, WHR, WHtR, FM, VF, AVI, and BAI in obese women^31^. In addition, Bodaghabadi et al.^32^ demonstrated that BMI, WC, HC, FM, and BAI decreased significantly in overweight women following a low-calorie diet.

BMI is commonly used for body weight categorization, but it does not exactly indicate the body composition and its measurement varies according to age, sex, and ethnic variations^33^. While, the WC, WHR, and WHtR are commonly used to evaluate central obesity^34,35^. WHtR is better than BMI in the prediction of DM and cardiovascular disease’s risks^36^. ABSI is a new body shape index which has been found to be more closely related with mortality risk than BMI and WC^37^. Moreover, ABSI has been suggested to possess a significant correlation with abdominal obesity^38^ and seems to predict mortality risk better than alternative indices of abdominal obesity^39^. AVI is also an anthropometric index for assessing central obesity and is associated with insulin resistance and DM^40^.

Recent Advancements in Medical Modeling: In recent years, there has been a growing trend toward mathematically modeling and analyzing various medical problems. Such mathematical models serve as invaluable tools for researchers, offering deeper insights into the underlying nature and mechanisms of diseases. By elucidating these complexities, mathematical models pave the way for the development of personalized treatment strategies, tailored to the unique characteristics of each patient. This individualized approach holds immense promise for optimizing treatment outcomes and patient satisfaction.

Furthermore, mathematical models play a crucial role in healthcare resource allocation and management. By accurately quantifying disease type and severity, these models enable healthcare professionals to make informed decisions regarding the allocation of medical resources, ensuring their optimal utilization. Moreover, by aiding physicians in precisely specifying disease types and severity, mathematical models facilitate the selection of the most appropriate treatment plans, thereby enhancing treatment efficacy and patient satisfaction.

The present study provided the mathematical equations as a quantitative representation of changes in anthropometric measurements in relation to weight changes. For example, according to our results the PC of WC was equal to: − 0.120 + 0.703 × PC-WT. It means that women under LCD experienced a decrease of about 0.7 cm in WC, for every 1 kg decrease in the PC-WT which was so promising for them. Similarly, the PC of HC was equal to: − 0.350 + 0.510 × PC-WT, which means that women under LCD experienced a decrease of about 0.5 cm in HC, for every 1 kg decrease in the PC-WT. Also, the PC of Body Fat was equal to: − 0.019 + 0.915 × PC-WT, which means that women under LCD experienced a decrease of about 0.9 unit in body fat percentage, for every 1 kg decrease in the PC-WT. Moreover, the PC of AVI was equal to: − 0.324 + 1.320 × PC-WT, which means that women under LCD experienced a decrease of about 1 unit in AVI, for every 1 kg decrease in the PC-WT. Additionally, the PC of ABSI and BAI was equal to: -0.112 + 0.034 × PC-WT and 0.002 +  − 0.002 × PC-WT, respectively, which means that women under LCD experienced a minimal change in ABSI and BAI for every 1 kg decrease in the PC-WT. Furthermore, the PC of WHtR was equal to: − 0.113 + 0.702 × PC-WT, which means that women under LCD experienced a decrease of about 0.7 unit in WHtR, for every 1 kg decrease in the PC-WT. Also, the PC of FFM was equal to: − 0.568 +  − 0.438 × PC-WT, which means that women under LCD experienced an increase of about 0.4 unit in FFM, for every 1 kg decrease in the PC-WT. Furthermore, the adjustment for age had minimal influence on the mathematical model utilized to assess the relationships between changes in body measurements over time and weight loss as the primary independent variable. This suggests that the integrity and robustness of the mathematical model were preserved, even with age taken into account.

Generally, the decreasing rates of WC, HC, Body Fat, WHtR, and AVI in relation to the weight loss were clinically and statistically significant. This means that a healthy weight lowering diet would be accompanied by decreasing body fat, body size and also the risk of morbidities.

To the best of our knowledge, there was only one study^24^ that reported the analytic correlation between weight loss and WC change as: % Weight loss = 0.85 × Waist reduction (cm) − 2.09; r = 0.79). This study also concluded that the reduction in WC, weight or BMI produced beneficial effects on certain cardiovascular risk factors, whereas reduction of WHR is less powerful predictor of risk factor improvements^24^.

Overall, the present study provides a new insight at the effects of a weight loss program as the prediction of variations in body size and anthropometric measurements in relation to weight changes. However, it has some limitations. We could not measure the blood pressure and any lab tests to determine the morbidity risk reduction following the weight loss. Moreover, all of our participants were women and therefore these results may not be generalizable to the men.

In conclusion, the present study showed a consistent decrease in all anthropometric measurements and fat mass of overweight/ obese women during five-months follow-up of LCD which were significantly associated with weight loss except than WHR. Moreover, the mathematical formulas reported in this study provide predictive relations and estimations of expected changes in various anthropometric indices based on the weight changes.

## Data Availability

The datasets used and/or analyzed during the current study available from the corresponding author on reasonable request.
